# Cerebral Abscess and epidural empyema in a young immunocompetent patient caused by *Streptococcus constellatus*

**DOI:** 10.1590/0037-8682-0031-2025

**Published:** 2025-07-07

**Authors:** Matheus Felipe Dantas Krause, Mariângela Ribeiro Resende, Gabriela Romantini Salioni, Vinicius de Menezes Jarry, Fabiano Reis

**Affiliations:** 1 Universidade Estadual de Campinas, Departamento de Radiologia e Oncologia, Campinas, SP, Brasil.; 2 Universidade Estadual de Campinas, Departamento de Clínica Médica, Campinas, SP, Brasil.

**Keywords:** Cerebral abscess, Streptococcus constellatus, Epidural empyema

## Abstract

*Streptococcus constellatus*, a member of the *Streptococcus anginosus* group, is a commensal bacterium that causes pyogenic infections, particularly abscesses. This report describes the case of a 28-year-old immunocompetent man who developed a brain abscess and epidural empyema after pneumonia. MRI suggested an abscess, which was confirmed by culture, revealing *S. constellatus*. Treatment included abscess drainage and ceftriaxone administration, which led to a full recovery. Diagnosis is challenging because of overlapping features with other bacteria. Imaging and culturing are crucial for identification. This case highlights the importance of considering *S. constellatus* in central nervous system infections, even in immunocompetent individuals.

## INTRODUCTION

*Streptococcus constellatus* is a gram-positive coccus belonging to the Streptococcus anginosus group (SAG), along with the bacteria *S. anginosus* and *S. intermedius*. Although considered a commensal organism of the human oral cavity and the gastrointestinal and genitourinary tracts^1^ ^,^ ^2^ ^,^ ^3^, *S. constellatus* is also known for its ability to generate pyogenic infections, particularly with the formation of abscesses^4^, both in immunosuppressed and immunocompetent patients.

Despite being present in the habitual bacterial flora of humans, *S. constellatus* presents a series of challenges in terms of diagnostic aspects because it requires a long culture time and its phenotypic elements overlap with those of the other members of the SAG^5^. Furthermore, regarding therapy, the need for a combination of drugs and surgical treatments is highlighted, in most cases, owing to the tendency of bacteria to form collections.

This report describes the case of an adult patient, immunocompetent and without comorbidities, who presented with headache, fever, and weight loss after recent pneumonia, whose diagnosis of brain abscess was suggested after brain magnetic resonance imaging (MRI) and confirmed after culture, which revealed the presence of *S. constellatus* within the lesion.

This case is noteworthy as it is the only case recorded in the last 25 years of a brain abscess associated with epidural empyema caused by *S. constellatus* in an immunocompetent adult, as illustrated by MRI. Other reported cases included only computed tomography (CT). A comprehensive search of the leading scientific databases (Cochrane Library, LILACS, SciELO, MEDLINE, PubMed, and PMC (PubMed Central) did not reveal any previously documented cases with the same clinical presentation, highlighting the rarity of this occurrence and the importance of reporting it to expand the current understanding of such infections.

## CASE REPORT

A 28-year-old man with no comorbidities, born and raised in Amparo, São Paulo, Brazil, was admitted for headache, mental confusion, memory loss, weight loss, nausea, vomiting, and fever for two weeks, approximately one month after being admitted to another service for pneumonia.

A physical examination revealed that the patient was conscious, oriented, and bradypsychic. His muscle strength was preserved, and the reflexes on the right side of the brain were slightly increased.

Brain CT revealed a hypodense lesion in the left frontal lobe with peripheral contrast enhancement and adjacent edema.

**FIGURE 1:**
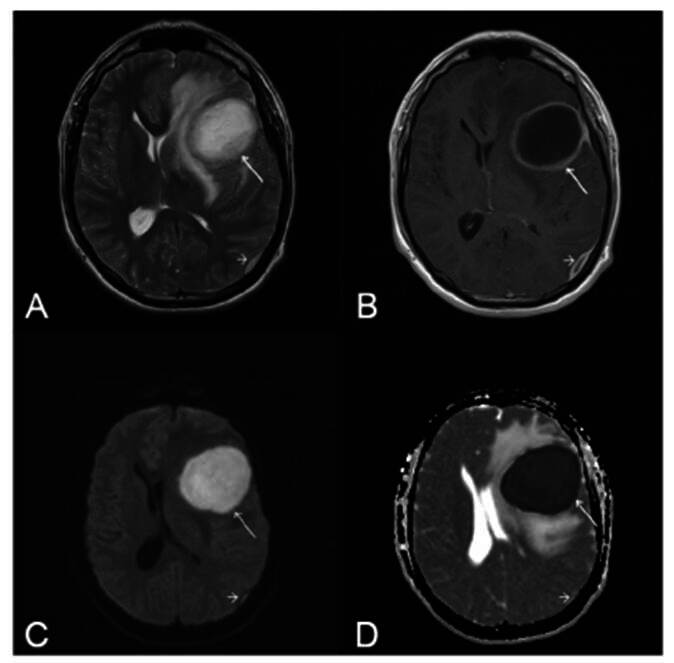
Axial T2 weighted image **(A).** T1 weighted image after contrast administration **(B).** Diffusion-weighted imaging (DWI) **(C).** Apparent diffusion coefficient (ADC) map **(D)**. These images show an expansible intracranial lesion in the left frontal lobe, with peripheral ring enhancement and perilesional edema (arrow) **(B)**, and restricted diffusion characterized by hyperintensity on DWI (arrow) **(C)** and hypointensity on the ADC map (arrow) **(D)**, as well as ipsilateral parieto-occipital epidural empyema **(short arrows in A, B, C, and D)**.

Brain MRI was performed (Figure **1**). It shows an expansile lesion in the left frontal lobe with peripheral contrast enhancement, restricted diffusion in the central core, and perilesional edema, suggestive of an abscess. In addition, an image of the ipsilateral parieto-occipital region suggested an epidural empyema.

After culturing the contents of the intracranial collection obtained by puncture via trepanation, the growth of *S. constellatus*, which is sensitive to penicillin, clindamycin, and vancomycin, was observed.

**FIGURE 2:**
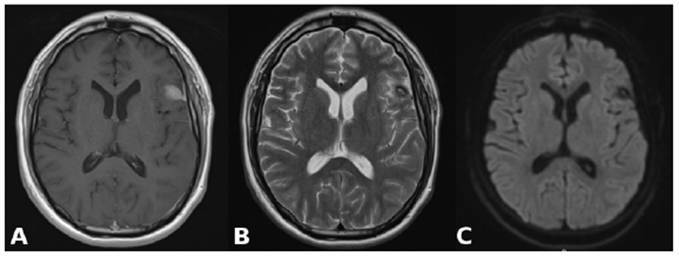
Axial T1 weighted image after contrast administration **(A)**. T2 weighted image **(B)**. Diffusion weighted (DW) image **(C)**. These images show that the expansible intracranial lesion in the left frontal lobe is diminished, with predominantly low signal intensity on a DW image; these findings indicate clear fluid in the abscess cavity.

Finally, after puncture drainage of the intracerebral abscess, treatment was completed clinically with ceftriaxone 4 g/day for two months, with complete improvement in the patient's systemic and neurological symptoms. A subsequent brain MRI scan (Figure 2) demonstrated that the intracranial lesion had diminished in size.

## DISCUSSION

Although it frequently colonizes the oropharyngeal and gastrointestinal tracts without causing disease, *S. constellatus*, similar to other GAS bacteria, can also cause serious invasive diseases^6^. 

In clinical practice, many infections caused by *S. constellatus* are not differentiated from those caused by other pyogenic agents, which may favor underdiagnosis of the agent. However, it is worth noting that its high virulence should be considered a potential etiological agent when in the presence of abscesses, notably in the head, neck, and abdomen, or when isolated in sterile sites.

Another important point regarding *S. constellatus* infections is the high prevalence of co-infection with anaerobic organisms, particularly those present in the oral cavity, such as *Fusobacterium sp.*, which often ends up being underdiagnosed owing to limitations related to culture techniques^7^.

Most reported cases of brain abscesses are related to*S. intermedius*, and only a few cases involving *S. constellatus*have been documented^8^. 

A CDC national call for cases identified 81 cases of pediatric abscesses and epidural and subdural empyemas, of which 61% had at least one respiratory infection, such as sinusitis (26%) and COVID-19 (18.2%). Subdural empyema was the most common presentation (53.1%), followed by brain abscesses (37.0%) and epidural empyema (33.3%). *S. intermedius*was the most frequently detected. The data indicated that streptococcal species were the most identified (92.1%), with S*. intermedius* (41.6%) and *Streptococcus anginosus* (18.4%) having the highest prevalence^9^. 

The clinical manifestations of brain abscesses are usually nonspecific. Symptoms of intracranial hypertension (increased intracranial pressure, headache, and visual perturbations), seizures, and focal neurological deficits are the most common presentations.

Imaging studies using CT and MRI are essential for the diagnosis. The MRI pattern is indicative of a pyogenic abscess, and the typical pattern includes single or multiple lesions with ring contrast enhancement and restricted diffusion in the core of the cavity, reflecting the presence of viscous materials, such as pus^10^.

It is worth noting that treatment generally consists of a combination of surgical intervention and antibiotic therapy since the bacteria usually cause abscess formation. Regarding the medications used, ceftriaxone is usually the main choice^11^ in association with some antibiotics with coverage against anaerobes, owing to the potential risk of coinfection^12^.

Initial treatment usually consists of ceftriaxone combined with metronidazole. An exception to this rule occurs when bacteremia or endocarditis is identified without collections visualized after imaging studies, in which case the coverage of anaerobes may be waived. Even so, it is worth noting that the main pillar of treatment is surgical intervention, and that the results of culture should ideally guide the choice of antibiotic therapy with an antibiogram.

Finally, this is the first case of brain abscess associated with epidural empyema caused by this microorganism in a patient with no known immunosuppression, as demonstrated by MRI, which emphasizes the importance of including *S. constellatus* among the etiological differential of pyogenic infections in general, notably of the central nervous system.
